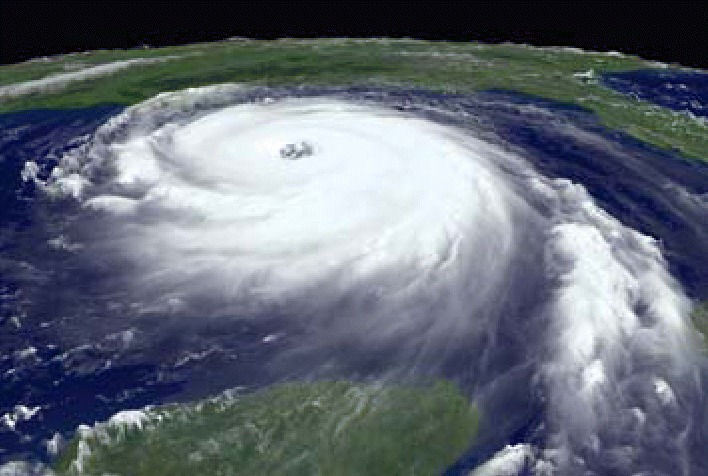# EHPnet: CDC: Environmental Concerns After Hurricane Katrina NIEHS: Natural Disaster Response

**Published:** 2006-01

**Authors:** Erin E. Dooley

Since Hurricane Katrina struck the U.S. Gulf Coast on 29 August 2005, Americans have sought reliable information on how to safely reenter flood-damaged environments. The U.S. Department of Health and Human Services (DHHS) has been at the forefront of the effort to assist those affected by this disaster. Two DHHS agencies, the Centers for Disease Control and Prevention (CDC) and the NIEHS, have developed websites offering information on dealing with post-hurricane conditions.

The CDC page, located at **http://www.bt.cdc.gov/disasters/hurricanes/environmental.asp**, gives visitors access to information from both the CDC and the U.S. Environmental Protection Agency (EPA). The site contains a 38-page report, released on September 17, summarizing an environmental health needs and habitability assessment of the city of New Orleans conducted by these two agencies. The report provides conclusions about the habitability of the city as well as recommendations on how best to go about allowing citizens to repopulate the city. There is also a health consultation on the Murphy Oil Company spill, which released 25,110 barrels of mixed crude oil into the area around Meraux and Chalmette, Louisiana.

The site also includes several documents to guide residents as they resume life along the Gulf Coast. There is basic information on cleaning up mold, disinfecting wells, protecting oneself from debris smoke, avoiding carbon monoxide, dealing with animal and insect hazards, and managing chemicals released during flooding. The mold cleanup section also links to other information sources, some of which are available in Spanish and Vietnamese (many Vietnamese have settled along the Gulf Coast since the 1950s). For response and cleanup workers there are links to federal guidelines and recommendations on personal protective equipment, cleaning HVAC systems, and handling and burning hurricane debris.

The NIEHS Natural Disaster Response page is located at **http://www-apps.niehs.nih.gov/katrina/**. The page features geographic information system (GIS) maps that the NIEHS and its academic partners created that identify chemical plants, refineries, Superfund sites, and other potential sources of contamination. It also contains satellite images of the areas affected by the hurricanes. In the future, the section will feature a functional set of GIS layers that will let visitors customize their own maps. These images can help decision makers and others in identifying sources and routes of contaminants, analyzing the potential for future exposures, assessing human exposures in the immediate aftermath of the hurricanes, and predicting long-term health impacts linked with these exposures.

The Questions and Answers page brings together resources from several federal agencies to answer frequently asked questions about mold, sewage, and seafood consumption. This page also contains information on the NIH Katrina Call Center, available at 1-866-887-2842, which provides round-the-clock medical consultation by telephone to anyone affected by Hurricane Katrina.

The NIEHS Program Resources section of the page has links to four programs that the NIEHS had in place long before the disaster struck, which are now being called into action. One of these, the Worker Education and Training Program, offers a PowerPoint presentation for cleanup workers titled *Protecting Yourself While Helping Others*, developed jointly by the NIEHS and other federal agencies to guide those responding to the storms of 2005. This presentation is also available in Spanish and Vietnamese. Visitors can also find safety posters for responders, guidelines for the protection and training of mold cleanup workers, and other checklists, safety plans, and materials.

As a service to NIH- and NIEHS-funded researchers at flooded universities, this site provides links to information for grantees affected by Hurricane Katrina, including notices from the *NIH Guide*.

## Figures and Tables

**Figure:**